# Role of medical imaging for immune checkpoint blockade therapy: From response assessment to prognosis prediction

**DOI:** 10.1002/cam4.2464

**Published:** 2019-08-05

**Authors:** Hong Wei, Hanyu Jiang, Bin Song

**Affiliations:** ^1^ Department of Radiology Sichuan University West China Hospital Chengdu Sichuan Province China

**Keywords:** biomarker, cancer, imaging, immune checkpoint blockade, response assessment

## Abstract

Immune checkpoint blockade (ICB) represents a promising approach in cancer therapy. Owing to the peculiar biologic mechanisms of anticancer activity, checkpoint blockers are accompanied with distinctive response patterns and toxicity profiles. Medical imaging is the cornerstone for response assessment to immunotherapy and plays a critical role in monitoring of immune‐related adverse events (irAEs). Imaging‐based biomarkers have shown tremendous potential for the prediction of therapeutic efficacies and clinical outcomes in patients treated with checkpoint inhibitors. In this article, the landscape of current response assessment systems for immunotherapy was reviewed with a special focus on the latest advances in the assessment of responses to ICB. Emerging imaging biomarkers were discussed along with the challenges regarding their clinical transformation. In addition, the biological mechanisms and clinical applications of ICB and irAEs were also within the scope of this review.

## Graphical abstract

Three atypical response patterns including pseudoprogression, dissociated response, and hyperprogression are accompanied with distinguished prognosis and pose significant challenges to response assessment to immunotherapy. A wide range of imaging biomarkers including anatomic, metabolic and functional parameters, radiomic features, and molecular probes have demonstrated enormous potential for predicting responses and prognosis in patients receiving immune checkpoint blockade therapy. Further efforts are warranted to achieve the clinical transformation of these predictive imaging biomarkers for optimizing patient selection and guiding the decision‐making process in routine practice.

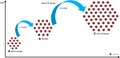

## 1 INTRODUCTION

In recent years, tremendous progresses have been achieved in cancer immunotherapy since the introduction of immune checkpoint blockade (ICB) to oncological practice.^1^, ^2^, ^3^ Immune checkpoint blockade represents a range of anti‐checkpoint monoclonal antibodies that could yield remarkable and durable anticancer responses (eg, overall survival [OS] >3 years).^4^, ^5^, ^6^, ^7^ Nevertheless, the response rate of ICB varies across diverse tumor types. In patients treated with anti‐CTLA‐4 monotherapy, the objective response rate (ORR) varied from 19% to 32% for melanoma.^8^, ^9^ In patients receiving anti‐PD‐1 monotherapy, the reported ORR of melanoma, non‐small cell lung cancer (NSCLC), renal cell carcinoma (RCC) and squamous cell carcinoma of the head and neck (SCCHN) was 45%, 39%, 25%, and 15%, respectively.^8^, ^10^, ^11^, ^12^ In addition, immune‐mediated complications have been observed during ICB therapy.^13^ In this scenario, accurate assessment of therapeutic efficacies and identification of robust biomarkers for potential responders are of clinical significance in optimizing patient management and guiding the decision‐making process in routine practice.

Medical imaging is the cornerstone for capturing tumor response or progression in immunotherapy. Currently, conventional computed tomography (CT) and magnetic resonance (MR) imaging are widely employed for the measurement of tumor burden by directly revealing morphological changes in target lesions.^14^ Furthermore, several emerging imaging modalities, including metabolic imaging, functional MR imaging, radiomics and molecular imaging, can provide important information regarding tumor biological behaviors and peritumoral microenvironment.^15^, ^16^, ^17^ Accordingly, imaging‐based biomarkers have shown tremendous potential to predict clinical responses and prognosis of cancer patients receiving ICB therapy.

In this article, we provided a landscape of current immune‐related response assessment systems, with a special focus on the latest advances and challenges in the assessment of responses to ICB therapy. We also discussed a wide range of emerging imaging biomarkers particularly for anti‐checkpoint therapy along with the challenges encountered in their clinical transformation. In addition, the biological mechanisms and clinical indications of checkpoint inhibitors and immune‐related adverse events (irAEs) were also described in this review.

## 2 IMMUNE CHECKPOINT BLOCKADE: A NEW “ACTIVATOR” IN CANCER IMMUNOTHERAPY

In the endogenous antitumor activities of immune system, T lymphocytes are the major effectors responsible for antigen recognition and initiation of antineoplastic activities.^1^ Immune checkpoints, a group of paired receptor‐ligand molecules, are capable of putting the brakes on CD8+ T lymphocytes to avert self‐tolerance and tissue damages in cases of pathogenic infection.^1^ Tumor cells could also dysregulate the activity of T cells and generate tumor immune resistance by activating certain immune checkpoint pathways.^1^, ^4^ Immune checkpoint inhibitors are monoclonal antibodies that could refresh the inactive T cells and unleash durable antitumoral responses by blocking various immune checkpoints, such as cytotoxic T cell lymphocyte‐associated protein 4 (CTLA‐4), and programmed death receptor 1 (PD‐1) and its ligand, known as programmed death receptor ligand 1 (PD‐L1).^1^, ^2^, ^3^, ^4^ Currently, six monoclonal antibodies targeting checkpoints have been approved by the United States Food and Drug Administration (FDA).^18^ The approved indications for these novel agents are described in Table 1.

**Table 1:** The United States FDA approved immune checkpoint inhibitors and their indications in routine practice

Category of ICIs	Brand name	Approved indications
Monotherapy		
CTLA‐4 blockade		
Ipilimumab	YERVOY	Melanoma
PD‐1 blockade		
Nivolumab	OPDIVO	Melanoma, NSCLC, SCLC, SCCHN, HCC, RCC, UCC, MSI‐H, and dMMR CRC, classic Hodgkin's lymphoma
Pembrolizumab	KEYTRUDA	Melanoma, NSCLC, SCCHN, cervical cancer, gastric cancer, HCC, MCC, UCC, MSI‐H, or dMMR solid tumors, MSI‐H or dMMR CRC, classic Hodgkin's lymphoma, primary mediastinal large B‐cell lymphoma
PD‐L1 blockade		
Atezolizumab	TECENTRIQ	UCC, NSCLC, ES‐SCLC, triple‐negative breast cancer
Durvalumab	IMFINZI	UCC, NSCLC
Avelumab	BAVENCIO	MCC, UCC
Combined therapy		
Ipilimumab + Nivolumab	YERVOY + OPDIVO	Melanoma, RCC, MSI‐H or dMMR mCRC

Note

The most recent update was on 18 April 2019.

Abbreviations: CRC, colorectal cancer; CTLA‐4, cytotoxic T‐lymphocyte antigen 4; dMMR, mismatch repair deficient; ES‐SCLC, extensive‐stage small cell lung cancer; FDA, Food and Drug Administration; HCC, hepatocellular carcinoma; MCC, merkel cell carcinoma; mCRC, metastatic colorectal cancer; MSI‐H, microsatellite instability‐high; NSCLC, non‐small cell lung cancer; PD‐1, programmed cell death protein 1; PD‐L1, programmed cell death protein ligand 1; RCC, renal cell carcinoma; SCCHN, squamous cell carcinoma of the head and neck; SCLC, small cell lung cancer; UCC, urothelial carcinoma.

## 3 THE ROLE OF MEDICAL IMAGING IN IMMUNOTHERAPY: RESPONSE ASSESSMENT AND TOXICITY SURVEILLANCE

In routine oncological practice, conventional imaging studies including CT, MR imaging, and positron emission tomography/computed tomography (PET/CT) play crucial parts in assessing immunotherapeutic effects and monitoring immune‐related toxicities. This section will review (a) the atypical response patterns observed in immunotherapy; (b) the landscape of current immune‐related response assessment systems; (c) the latest observations and challenges in the radiological assessment of immunotherapy; (d) the imaging patterns of common irAEs; and (f) the application of medical imaging across diverse tumor types.

### 3.1 Atypical response patterns in immunotherapy

Owing to the peculiar biologic mechanisms, immune checkpoint blockades are accompanied with atypical response patterns, such as pseudoprogression, mixed response (or dissociated response) and, more recently reported, hyperprogressive disease (HPD).^19^, ^20^

#### 3.1.1 Pseudoprogression

**Figure 1:**
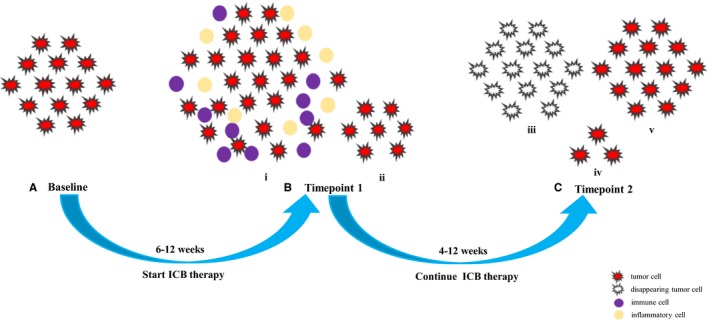
Schematics of pseudoprogression. For capturing of pseudoprogression, one imaging study before immune checkpoint blockade (ICB) therapy (A) and at least two imaging scans after treatment (B, C) are required. Pseudoprogression is shown as the enlargement of preexisting lesions (i) and/or the appearance of new lesions, (ii) followed by a decrease in tumor burden, which can be manifested as the disappearance of index lesions, (iii) shrinkage, (iv) or durable stable disease, (v) time intervals vary relying on the tumor types, agents and treatment strategies. In most cases, baseline to timepoint 1 is 6‐12 wk, and timepoint 1 to timepoint 2 is 4‐12 wk

Pseudoprogression is described as an evidence of radiological progression, including tumor enlargement and/or the appearance of new lesions, followed by response or stabilization on follow‐up imaging (Figure 1).^18^, ^23^ The incidence of pseudoprogression is relatively low (~10% in melanoma; ~5% in NSCLC),^6^, ^21^, ^22^, ^23^, ^24^ which indicates that a considerable portion of the suspected pseudoprogressors might have true progressive diseases (PDs).^23^ However, patients experiencing pseudoprogression can achieve sustained clinical benefit and have favorable prognosis compared with true progressors.^21^ To reduce inappropriate withdrawal of treatment, longitudinal imaging scans for monitoring of tumor burden dynamics are warranted to capture this unique response pattern. The phenomenon of psuedoprogression probably results from the ongoing tumor growth before the achievement of adequate immune response, the cytotoxic T lymphocytes (CTLs) recruitments and inflammatory responses in tumor milieu.^26^, ^27^

#### 3.1.2 Dissociated response

**Figure 2:**
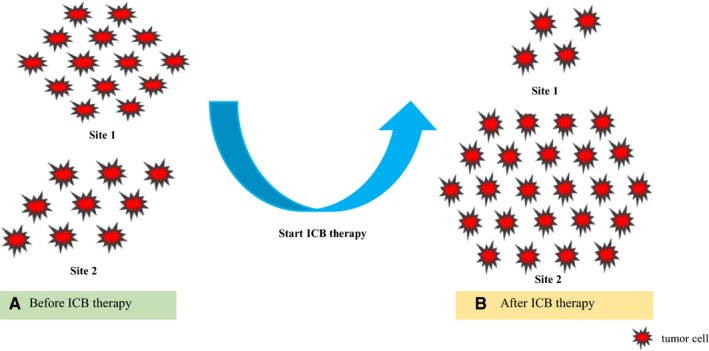
Schematic diagrams of dissociated response. For radiological evaluation, one imaging study before immune checkpoint blockade (ICB) therapy (A) and at least one imaging scan after treatment (B) are required. Dissociated response describes disparate responses across different organs at the same time. As is shown in the graphs, an obvious shrinkage can be observed in site 1 (A, B), whereas an increase in tumor burden is presented in site 2 (A, B) after ICB therapy. However, through the whole‐body tumor burden imaging assessment, we can observe that the whole‐body tumor burden after ICB therapy (B) is increased compared with that before treatment (A), which may indicate poor clinical outcomes

Dissociated response, namely, mixed response, refers to concomitant shrinkage of some lesions and flare‐up of others, indicating disparate responses across different organs (Figure 2).^28^, ^29^ In a recent study of 160 NSCLC patients treated with anti‐PD‐1/PD‐L1 therapy, dissociated responses were observed in 12 patients (7.5%); of whom six (50%) patients obtained clinical benefit, whereas the remaining patients presented with tumor progression.^29^ In another study of 166 NSCLC patients receiving anti‐PD‐1 therapy, one patient demonstrated mixed response with a dramatic decline in primary tumor burden but a substantial enlargement of right supraclavicular lymph node, and the latter turned out to be a metastasis.^23^ On the basis of these observations, the prognosis of patients with dissociated response is indeterminable. Comprehensive evaluations integrating whole‐body tumor burden imaging and patient clinical status are warranted to better appraise the patient's clinical responses. Of note, dissociated response is an atypical response pattern but not exclusively restricted to immunotherapy. It is unclear that what are the links or key factors for triggering this atypical pattern during immunotherapy. The mechanisms of dissociated response remain to be illuminated in the future.

#### 3.1.3 Hyperprogressive disease

**Figure 3:**
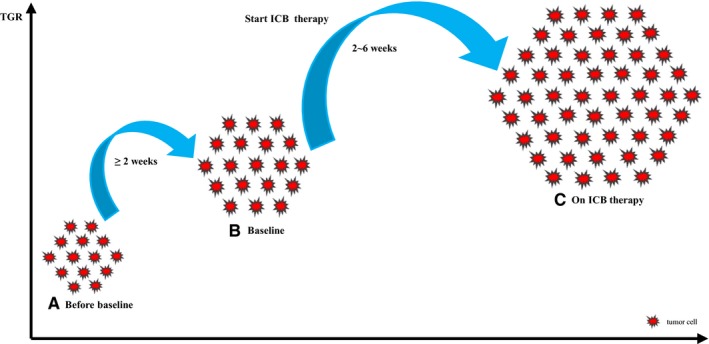
A graphical representation of hyperprogressive disease. For radiological assessment, at least two computed tomography (CT) scans before immune checkpoint blockade (ICB) therapy—baseline (B) and the most recent scan before baseline (A) and one CT scan during treatment (C) are required. Hyperprogressive disease is defined as progressive disease per RECIST 1.1 at the first CT scan and ΔTGR exceeding 50%. Time interval between CT scans should not be less than 2 wks, and baseline CT scan must be conducted within 6 wk before ICB therapy initiation. This graph was modified from reference 24. RECIST, response evaluation criteria in solid tumors

Hyperprogressive disease refers to a remarkably accelerated tumor growth rate (TGR) that leads to poor prognosis in patients under ICB therapy particularly anti‐PD‐1/PD‐L1 agents.^24^, ^25^, ^30^, ^31^, ^32^ Currently, no consensus has been reached on the definition of HPD.^24^ For instance, Champiat et al first reported HPD as a RECIST 1.1 progression before ICB therapy and a twofold or greater increase in TGR during immunotherapy,^30^ whereas Ferrar et al defined HPD as a disease progression at the first evaluation with at least an increase of 50% ΔTGR (the variation of TGR before and on treatment) (Figure 3).^24^ The incidence of HPD was reported to range from 7% to 29% across different types of carcinoma.^24^, ^25^, ^30^, ^31^, ^32^ However, the discordance in the definition of HPD across published literatures challenges the direct comparison of data.

An increasing number of studies focused on this new atypical pattern in ICB therapy, several indicators had been reported to be correlated with HPD. Champiat et al found that hyperprogression was associated with older age (≥65 years old) and worse OS in a cohort with 131 patients with various tumor types treated with monotherapy by anti‐PD‐1/PD‐L1 antibodies.^30^ Notably, they found that HPD was not related with tumor burden at baseline or tumor type. More recently, Kanjanapan et al analyzed data from 182 patients with mixed solid tumors receiving single‐agent or combination immunotherapy adopting the same methodology used by Champiat et al.^32^ Intriguingly, they found that HPD was not associated with age but sex—a higher incidence of HPD was observed in females compared with males, whereas age, clinically significant adverse events, tumor type, and the type of immunotherapy did not show significant differences between hyperprogressors and non‐hyperprogressors. Another multicenter study including 406 NSCLC patients by Ferrar et al, the largest research investigating HPD to date, revealed that HPD was associated with poor prognosis and higher metastatic burden before treatment (>2 metastatic sites).^24^ Other factors have also been reported to be correlated with HPD, including locoregional recurrence, liver metastases, ECOG performance status (PS) of 1 or 2, a larger sum of diameters of target lesions at baseline, and elevation in absolute neutrophil count (ANC) and C‐reactive protein (CRP) levels in the first 4 weeks.^25^, ^31^ However, further efforts are required to validate these findings and figure out the underlying mechanisms of HPD.

In summary, the phenomena of these atypical responses under ICB therapy appears to be restricted to a small fraction of patients. Pseudoprogression is associated with delayed and durable clinical benefits, prognosis of dissociated response is inconclusive, whereas hyperprogression is related to dismal clinical outcomes. These atypical response patterns complicate the patient management in routine practice. Medical imaging plays a crucial part in longitudinal monitoring of tumor burden changes in immunotherapy. Future studies are warranted to explore the predictive biomarkers for these atypical responses, which could be beneficial to patient stratification and precise therapeutic decision‐making.

### 3.2 The landscape of immune‐related response assessment systems

Historically, World Health Organization (WHO) criteria and Response Evaluation Criteria in Solid Tumors (RECIST) have provided standardized guidelines in the response assessment of chemotherapy. However, neither criteria are able to capture pseudoprogression in immunotherapy. To provide an accurate classification of tumor responses, several immune‐related standard systems were proposed successively, including immune‐related response criteria (irRC), immune‐related RECIST (irRECIST), and immune RECIST (iRECIST) (Table 2).

**Table 2:** Comparison between conventional criteria and immune‐related response assessment systems

	WHO (1979)	irRC (2009)	RECIST1.0 (2000)	RECIST1.1 (2009)	irRECIST (2013)	iRECIST (2017)
Measurements	BD	BD	UD	UD	UD	UD
New, measurable lesions	Designates PD	Incorporates into TTB for the assessment of PD	Designates PD	Designates PD	Incorporates into TTB for the assessment of PD	Separately documents; adds into the assessment of PD
CR	Disappearance of all IL and NIL Nodal short axis diameter <10 mm No new lesions					
PR^a^	≥50% decrease in TB, non‐unequivocal progression for NIL or no new lesions	≥50% decrease in TTB, non‐unequivocal progression for NIL or no new lesions	≥30% decrease in TB, non‐unequivocal progression for NIL or no new lesions	≥30% decrease in TB, non‐unequivocal progression for NIL or no new lesions	≥30% decrease in TTB, non‐unequivocal progression for NIL or no new lesions	≥30% decrease in TB, non‐unequivocal progression for NIL or no new lesions
SD	Neither PR nor PD					
PD^b^	≥25% increase in TB and/or progression of NIL and/or new lesions	≥25% increase in TTB	≥20% increase in TB and/or progression of NIL and/or new lesions	≥20% increase in TB and ≥5 mm increase in absolute value, and/or progression of NIL and/or new lesions	≥20% increase in TTB and ≥5 mm increase in absolute value, and/or progression of NIL and/or new lesions	iUPD—≥20% increase in TB and/or progression of NIL and/or new lesions
Confirmation of PD	NA	≥25% increase in TTB	NA	NA	New unequivocal progression or continued progression from the first PD; presence of another new lesions	iCPD—≥5 mm increase in new IL, increased size of IL or NIL, progression of new NIL, presence of another new lesions

Abbreviations: BD, bidimensional (longest diameter × longest perpendicular diameter for both nonnodal lesions and lymph nodes); CR, complete response; iCPD, immune‐confirmed progressive disease; IL, index lesions; iRECIST, immune RECIST; irRC, immune‐related response criteria; irRECIST, immune‐related RECIST; iUPD, immune‐unconfirmed progressive disease; NA, not available; NIL, non‐index lesions; PD, progressive disease; PR, partial response; SD, stable disease; RECIST, response evaluation criteria in solid tumors; TB, tumor burden; TTB, total tumor burden; UD, unidimensional (longest diameter for nonnodal lesions, longest perpendicular diameter for lymph nodes); WHO, World Health Organization.

a In reference to the baseline.

b In reference to the nadir (minimum recorded TB).

#### 3.2.1 Immune‐related response criteria

Immune‐related response criteria were the first immune‐adapted criteria proposed in 2009, which were modified on the basis of WHO criteria.^28^ The major innovations of irRC included: (a) new measurable lesions were incorporated into the total tumor burden (TTB) for response evaluation; and (b) except for immune‐related stable disease (irSD), the designation of immune‐related complete response (irCR), immune‐related partial response (irPR), and particularly immune‐related PD (irPD) required confirmation with a repeat and consecutive imaging scan at least 4 weeks from the date first recorded.^28^ Notably, the confirmation of PD was designed to capture pseudoprogression. Nevertheless, some disadvantages including bidimensional measurements and multiple index lesions to be measured limit its wide use in practice.

#### 3.2.2 Immune‐related RECIST

In 2013 and 2014, Nishino et al successively proposed irRECIST to remedy the limitations of irRC.^33^, ^34^ First, irRECIST simplified the measurement of tumor burden by allowing unidimensional measurements instead of bidimensional measurements. Second, irRECIST reduced the number of target lesions to be evaluated (Table 2). However, key features of irRC were maintained in irRECIST, such as inclusion of new lesions into TTB measurement and confirmation of PD.^33^, ^34^, ^35^ Notably, in irRECIST, confirmation of progression was not mandatory but recommended for patients with an increase of TTB ≥20% particularly within 12 weeks after the treatment onset to avoid the loss of salvage therapies.^33^, ^36^

#### 3.2.3 Immune RECIST

To ensure consistent study design and data collection and facilitate the ongoing collection of trial data, iRECIST was proposed by RECIST working group in 2017, designed to integrate modified RECIST (RECIST version 1.1) into cancer immune‐related criteria.^37^ There were two major innovations in iRECIST. First, new lesions were separately documented and added into the assessment of progression. Second**,** a novel concept known as immune unconfirmed PD (iUPD) was proposed. Immune unconfirmed PD refers to an initial RECIST 1.1 PD that still requires to be confirmed. Immune‐unconfirmed PD could be reassigned a couple of times so long as immune‐confirmed PD (iCPD) has not been reached. Immune‐confirmed PD should be evaluated at follow‐up imaging in 4‐8 weeks after iUPD, described as the presence of additional new lesions or a further increase in tumor burden compared with iUPD. To note, iRECIST is not expected to be a guidance of therapeutic decision‐making in routine clinical practice; all decisions about continuation or termination of treatment should be codetermined by multidisciplinary discussions with oncologists and patients.^19^, ^37^, ^38^

In summary, immune‐adapted systems could capture responses otherwise missed with RECIST in a small number of patients.^21^, ^23^, ^28^, ^29^, ^39^, ^40^ However, these novel criteria should be considered as indispensable complements but not alternatives to conventional RECIST. Because pseudoprogression is an uncommon occurrence and clinical practice might not change accordingly. Furthermore, iRECIST were derived from expert consensus, further efforts are required to validate the superiority and applicability of this novel system.^41^

### 3.3 New observations and challenges in the radiological assessment of immunotherapy

#### 3.3.1 Symptomatic pseudoprogression

Immune RECIST might fail to capture pseudoprogression, particularly in patients with “symptomatic pseudoprogression.” In general, pseudoprogression should not be accompanied with the deterioration of clinical status, which usually indicates the true progression. However, a recent review by Vrankar et al reported the “symptomatic pseudoprogression” in eight NSCLC patients treated with anti‐PD‐1/PD‐L1 therapy.^42^ These eight patients experienced psuedoprogression accompanied with clinical deterioration, followed by clinical benefit after 4‐20 weeks after treatment onset. According to iRECIST, treatment beyond progression per RECIST 1.1 should only be allowed in patients with a stable clinical status.^37^ However, due to the lack of alternative salvage therapies, these patients continued to receive anti‐checkpoint agents and ultimately benefited from the continuation of immunotherapy.

Owing to insufficient evidence, the underlying mechanisms of the symptomatic pseudoprogression are unclear currently. However, its existence further complicates the situation of immunotherapeutic response assessment. Regardless, it reminds clinicians that decision‐making regarding a sustained or terminated therapy should rely on the adequate consideration of the patient's clinical status, treatment response patterns, and available salvage therapies.

#### 3.3.2 Hyperprogression

The current immune criteria might fail to enable an early detection of HPD during ICB therapy. Ferrar et al defined HPD as RECIST 1.1 progression at the first CT evaluation and ΔTGR exceeding 50%.^24^ TGR was defined as a percentage increase in tumor volume within 1 month.^24^ ΔTGR was the variation in TGR upon and before treatment, allowing the evaluation of the relationship between treatment and tumor growth.^24^ Therefore, the radiological assessment of HPD requires at least two imaging studies before immunotherapy (baseline and the most recent study before baseline) and one imaging study during treatment.^20^, ^24^ However, iRECIST did not incorporate the pretreatment imaging data into the determination of TGR. Hence, HPD can only be detected at the second imaging assessment after treatment initiation.^20^ According to iRECIST, the RECIST PD was defined as iUPD at the first imaging assessment (8‐12 weeks after therapy onset), and iCPD was determined in 4‐8 weeks after iUPD.^37^ Using iRECIST, patients with early HPD need to continue ineffective therapies for at least 12 weeks before HPD is identified.^20^ Therefore, conducting an early imaging assessment (eg, at first 4‐6 weeks after treatment onset) and integrating the pretreatment tumor kinetics are mandatory for an early detection of HPD in patients receiving anti‐checkpoint therapy.

### 3.4 Immune‐related adverse events

Despite the increased response rate and prolonged survival, ICB is accompanied with a wide spectrum of toxicities, known as irAEs, which frequently involve in gastrointestinal tract, endocrine glands, skin, and liver.^13^ Less common sites involved are central nervous system, cardiovascular system, lungs, skeletal musculature, etc.^13^ Beyond therapeutic response assessment, medical imaging also plays an indispensable role in the detection and monitoring of irAEs. As described in Table 3, the most common sites and imaging characteristics of irAEs were summarized. The optimal imaging tools for the detection of each type of irAEs were also recommended. These data might help oncologists and radiologists to be familiar with the common irAEs, thus increasing the detection rate of immune‐mediated complications. It must be highlighted that optimal management of irAEs should be established on the comprehensive evidence of imaging findings, biochemical results, and clinical presentations.

**Table 3:** Common sites and imaging patterns of irAEs in immune checkpoint blockade therapy

Systems	irAEs	Imaging patterns	US	CT	MRI	PET
Endocrine system	Hypophysitis	Diffuse pituitary enlargement, stalk thickening, no compression of the optic apparatus, homogeneous or heterogeneous patterns of enhancement and intense ^18^F‐FDG uptake^43^, ^45^		±	+++	+
	Thyroiditis	Thyroid gland enlargement, hypoechoic and heterogenous echotexture on US, heterogeneous enhancement and intense and diffuse ^18^F‐FDG uptake^43^, ^46^	++			+
Respiratory and circulatory system	Pneumonitis	Interstitial pneumonia: (a) cryptogenic organizing pneumonia (COP)‐like pattern; (b) ground glass opacities (GGO); (c) sarcoid‐like pattern (hilar lymphadenopathy with or without micronodules, GGO and peribronchial interstitial thickening prevalent in hilar regions)^47^		+++		+
	Sarcoid‐like lymphadenopathy	Mediastinal and hilar lymphadenopathy with or without pulmonary changes (ie, nodular thickening of peribronchovascular bundles and the interlobular septum) and moderate uptake of ^18^F‐FDG^18^, ^43^		++		++
Digestive system	Colitis^44^	(a) Diffuse colitis pattern; (b) segmental colitis accompanied with diverticulosis pattern; (c) isolated recto‐sigmoid colitis without diverticulosis pattern. (ie, mesenteric vessel hyperemia, mild diffuse/segmental colonic wall thickening, fluid‐filled distended colon, pericolic fat stranding, and diffuse/segmental colonic mucosal hyperenhancement )^46^, ^48^, ^49^		++		+
	Hepatitis	Hepatomegaly, diffuse low‐attenuation of liver parenchyma on CT, periportal/gallbladder edema, periportal lymphadenopathy, and heterogeneous parenchymal enhancement with low‐attenuation areas on CT^43^, ^46^, ^50^	+	++	+++	±
	Pancreatitis	Focal or diffuse pancreatic enlargement, peripancreatic fat stranding on CT, reduced enhancement, and diffuse increased ^18^F‐FDG uptake^51^		++	+++	+
Musculoskeletal system	Arthritis	Soft tissue swelling, synovitis, and subchondral erosions^43^	++		+++	+

Note

This table was modified according to reference 43.

Abbreviations: CT, computed tomography; ^18^F‐FDG, ^18^F‐fluorodeoxyglucose; irAEs, immune‐related adverse events; MRI, magnetic resonance imaging; PET, positron emission tomography; US, ultrasound.

### 3.5 Application of medical imaging across diverse tumor types

Within cancer imaging, anatomical imaging by CT and MR imaging is currently applied in standard clinical practice for detection, characterization, staging, therapeutic efficacy assessment, and recurrence and metastasis surveillance of various malignancies. For example, high‐resolution CT is frequently used for the screening of single pulmonary nodule, whereas contrast‐enhanced MR imaging is commonly applied to detect brain metastases. In addition, functional imaging provides added information regarding tumor biological behaviors by revealing changes at the molecular, cellular, and organizational levels. For instance, dynamic contrast‐enhanced MR imaging (DCE‐MRI) can be used to investigate the relationship between tumor microvascularity and histopathological grade, tendency for metastasis and recurrence and patient prognosis, etc.^56^ Diffusion‐weighted imaging (DWI) can be applied to characterize tumors and identify early changes after treatment intervention. Furthermore, ^18^F‐fluorodeoxyglucose (^18^F‐FDG) PET provides complementary information to structural imaging techniques by uncovering tumor metabolic changes,^57^ which is generally used for the diagnosis and staging of tumors with aggressiveness and metabolic activity, such as melanoma, lymphoma, and lung, breast, esophageal, colorectal, or head and neck cancer.^56^

In summary, anatomic imaging is the most frequently applied imaging tool in clinical practice for the management of cancer patients. Nevertheless, structure imaging alone is inadequate to fully characterize and monitor tumor lesions.^56^ Integrating functional and metabolic imaging modalities into the anatomic imaging techniques contributes to maximize the value of information acquired for tumor detection, characterization, and surveillance.^56^

## 4 IMAGING BIOMARKERS FOR IMMUNE CHECKPOINT THERAPY

In view of the limited response rate and immune‐induced toxicities, identification of robust biomarkers before treatment is critical to determine the best candidates for ICB therapy. Currently, a wide range of biomarkers have been under investigation, of which most were derived from tissue or blood.^52^ However, some limitations are prominent in each type of biomarkers. First, tissue‐based biomarkers, such as PD‐L1 expression, tumor infiltrating lymphocytes, mutational or neoantigen burden,^53^ are often evaluated using invasive histopathological tests and demonstrate restricted capacity in capturing the spatial and temporal heterogeneity of tumors.^52^ Second, noninvasive blood‐based biomarkers, that is, the account of lymphocytes and other immune cells circulating, peripheral blood T cell subsets and liquid biopsy,^54^ cannot provide accurate information on the status of each individual tumor lesion.^52^ Given these unmet clinical needs, noninvasive imaging biomarkers that allow assessment of both tumor lesions and the surrounding microenvironment may serve as highly valuable complements to tissue and blood biomarkers.^16^

### 4.1 Anatomic imaging

Tumor burden used to be the most important surrogate in the therapeutic effect assessment by size‐based criteria. Tumor density can reflect the intratumoral vasculature or necrosis that might be of predictive value.^40^ Recently, several studies consistently found that the changes in tumor burden might serve as predictive biomarkers for clinical outcomes in advanced cancer patients receiving immunotherapy.^22^, ^40^, ^55^ Nishino et al found that an increase of tumor burden per irRECIST less than 20% from the baseline CT scans was associated with longer OS (*P* < .01) in advanced melanoma patients during pembrolizumab treatment.^22^ Dercle et al reported that high tumor burden, defined as tumor burden per RECIST 1.1 >9 cm, was an independent predictor of OS (*P* < .01) in patients with various tumor types treated with anti‐PD‐1/anti‐PD‐L1 therapy.^55^ Similarly, another study by Khoja et al comparing four radiological criteria (RECIST 1.1, irRC, CHOI and mCHOI) showed that changes in tumor size and density might be independent biomarkers for predicting OS (*P* < .01) in patients with metastatic melanoma treated with pembrolizumab.^40^

These preliminary observations proposed a practical noninvasive biomarker for predicting clinical benefit of ICB therapy.^22^ However, the capacities of anatomic imaging in the characterization and monitoring of tumor evolution are limited.^55^, ^56^ In addition, no consensus has been reached on whether tumor burden could be a reliable biomarker in immunotherapy. Therefore, further large‐scale prospective studies are warranted to validate the efficacy of these biomarkers.

### 4.2 Metabolic imaging

^18^F‐FDG PET/CT scan can depict early changes in tumor metabolism prior to overt morphologic changes, which sparks a variety of studies to investigate the feasibility of ^18^F‐FDG PET/CT scan in predicting the treatment efficacy and survival benefit of patients under ICB therapy.^57^, ^59^ Cho et al proposed a novel criteria termed as PET/CT criteria for early prediction of response to ICI therapy (PEPRIT) based on functional and anatomic parameters derived from PET/CT scans at 3‐4 weeks during immunotherapy to predict treatment response at 4 month of ICB therapy in melanoma patients with a sensitivity of 100%, specificity of 93%, and accuracy of 95%.^57^ They also observed that early increased ^18^F‐FDG uptake at 3‐4 weeks during ICB therapy was likely associated with favorable clinical outcomes.^57^ Their findings suggested the occurrence of early inflammatory response at the tumor milieu was induced by ICB. Results from another trial involving 24 NSCLC patients receiving nivolumab showed that metabolic response evaluated by ^18^F‐FDG could accurately predict response and prognosis at 1 month after nivolumab therapy.^58^ Ito and colleagues found that tumor response on ^18^F‐FDG PET/CT scans according to PET response criteria in solid tumors (PERCIST) was significantly associated with OS in melanoma patients undergoing ipilimumab therapy.^60^ Meanwhile, in another study, they also revealed that tumor burden measured with whole‐body metabolic tumor volume (wMTV) can serve as a robust biomarker for predicting prognosis in melanoma patients treated with ipilimumab.^61^ According to their findings, patients with a wMTV more than 27 cm^3^ would have a shorter median OS time compared with those with a wMTV below that cutoff value (10.8 vs 26.0 months).

Outcomes from these studies indicated that ^18^F‐FDG PET/CT scan can provide complementary information to classic anatomic imaging including CT and MR imaging,^57^ however, larger cohorts are warranted to validate these findings from studies with small sample size. Of note, it is essential for radiologists to realize that increased ^18^F‐FDG uptake is not specific for neoplasms regardless of its high sensitivity, because other pathological processes, including inflammatory reaction, can also show elevated glucose metabolism. In this setting, it is a great challenge for readers to distinguish tumor progression and metastases from inflammation on PET scans.^54^, ^57^, ^62^ Future efforts are warranted to solve this dilemma in ^18^F‐FDG PET/CT.

### 4.3 Functional imaging

Diffusion‐weighted imaging permits the measurement of random translational diffusion of water molecules and therefore provides pivotal information on tissue cellularity and cytomembranes integrity.^63^ Apparent diffusion coefficient (ADC), a quantitative parameter derived from DWI, can reveal the magnitude of water molecule motion in the extracellular compartment.^64^ Malignancies may demonstrate restricted diffusion and lower ADC values because of their high cellularity, whereas tumors with increased diffusion and higher ADC values can be seen as the evidence of therapeutic benefit. This can be explained by the fact that inflammation and necrosis caused by effective treatment would lead to prominent extracellular edema and decreased cellularity. Qin et al enrolled 10 recurrent glioblastoma patients undergoing anti‐PD‐1 therapy and observed initial volumetric expansion in tumor lesion within 0‐6 months across different imaging sequences.^65^ Potential explanations for the early increase in tumor volume were continued tumor growth due to delayed responses to immunotherapy and the infiltration of inflammatory cells, which would also reduce the ADC values of the tumor lesions.^65^ They found that stable or improved intermediate ADC volumes of interest (IADC VOI) after 6 months of therapy onset can provide more useful predictive information on therapeutic benefit compared with conventional imaging, including contrast‐enhanced T1‐weighted imaging (T1WI), FLAIR‐T2–weighted imaging (T2WI) and response assessment for neuro‐oncology (RANO) criteria measures.^65^

Robust evidence in terms of the predictive value of DWI remains to be proposed. Likewise, the suboptimal image quality (eg, poor signal noise ratio, limited spatial resolution, and artifacts) and low reproducibility of ADC measurements may hinder the applicability of DWI in practice.^63^ Therefore, diffusion images are recommended to be interpreted in conjunction with conventional imaging sequences, such as T1WI, T2WI, and contrast‐enhanced sequences.^63^

### 4.4 Radiomics

Radiomics is an imaging processing and analysis technique that enables the conversion of routine radiological images into quantitative data and the subsequent mining of high‐dimensional data.^17^, ^66^ The assumption for radiomics is that biomedical imaging can reflect both macroscopic and pathophysiological characteristics of tissues, which can be uncovered by quantitative image analyses.^15^, ^17^ The ultimate goal of radiomics is to generate imaging‐based biomarkers to improve knowledge of tumor biological behaviors and advance the development of precision medicine.^66^

Several studies have demonstrated that CT‐based radiomics features might effectively predict responses and prognosis in patients treated with immunotherapies.^15^, ^67^, ^68^ Sun et al analyzed the data from four independent multicenter cohorts of 491 patients with advanced solid tumors who received anti‐PD‐1/PD‐L1 monotherapy and established a CT‐based radiomics signature of tumor‐infiltrating CD8 cells.^15^ The radiomics signature was comprised of eight variables and demonstrated good prediction performance for the gene expression signature of CD8 cells (area under the receiver‐operator characteristic [AUROC]: 0.67) and tumor immune phenotypes (AUROC: 0.76). They observed that a higher radiomic score at baseline was significantly associated with objective response, controlled disease, and favorable OS. More recently, Tunali and colleagues established clinical‐radiomic models to predict two rapid disease progression phenotypes (time to progression [TTP] <2 months and HPD) in a cohort of 228 NSCLC patients.^68^ In this study, radiomic features were extracted from both the intratumoral and peritumoral regions based on baseline contrast‐enhanced CT images within 1 month prior to the initiation of ICB therapy. In terms of TTP <2 months, the prediction model incorporated four clinical covariates (hepatic and bone metastasis, previous lines of systemic therapies, and neutrophils to lymphocytes ratio) and four radiomic features (radial gradient border SD‐2D, border quartile coefficient of dispersion, border 3D Laws E5E5L5, and 3D Laws E5L5E5) and revealed an AUROC of 0.804, specificity of 83.4%, sensitivity of 63.4%, and accuracy of 73.4%. Notably, three of the four radiomic features were extracted from the border regions of tumors, where appeared to be more informative than intratumoral regions in reflection of the heterogeneity of tumor microenvironment.^68^ With regard to HPD, the prediction model was based on Royal Marsden Hospital prognostic score and one radiomic feature derived from the tumor border (border neighborhood gray‐tone difference matrix strength) with an AUROC of 0.865, specificity of 92.9%, sensitivity of 74.0%, and accuracy of 82.3%. Likewise, Durot et al analyzed the texture features of 74 metastatic lesions on baseline contrast enhancement CT images in 31 patients with metastatic melanoma receiving pembrolizumab and revealed that tumor skewness at coarse texture scale was an independent predictor of OS and progression‐free survival.^67^

These pioneering efforts are of great significance in revealing a promising direction for radiomics‐based biomarkers in immunotherapy. However, some barriers, including the image acquisition variability, high‐probability of false‐positive results, overfitting, challenges in result interpretation, and low reproducibility, probably slow down the clinical transition of radiomics.^16^ Future prospective studies with rigorous designs and standardized imaging acquisitions are warranted to further investigate the capacities of radiomics in immuno‐oncology. Since both hyperprogression and pseudoprogression can be manifested as an accelerated tumor growth early during the course of immunotherapy, future studies are warranted to investigate the feasibility of radiomics in distinguishing these two atypical immune‐related response patterns.^32^ In addition, integration of radiomic features into clinical, histopathological, and genomic biomarkers might provide added information regarding more precise malignant biological property profiling for predictive purposes.^69^

### 4.5 Molecular imaging

The application of molecular imaging in immuno‐oncology is related to employing radiolabeled antibodies, antibodies fragments, peptides, and proteins to track immune checkpoint targets at the cellular and molecular levels and to uncover the dynamic changes of the biomarkers expression.^70^ Preclinical studies showed that immuno‐PET monitoring of CD8+ T cells could serve as a predictive and prognostic biomarker for immune checkpoint therapy.^71^, ^72^ Currently, effective efforts are underway to define PD‐L1 or PD‐1 expression using noninvasive molecular probes, of which a large number of studies focused on the feasibility of antibody‐based tracers in immunotherapy.^73^, ^74^, ^75^, ^76^, ^77^, ^78^, ^79^, ^80^, ^81^, ^82^

For PET/CT‐based molecular imaging, ^64^Cu and ^89^Zr are the most common radionuclides used in immuno‐oncology. For instance, Natarajan et al developed ^89^Zr‐keytruda and ^64^Cu‐keytruda to visualize PD‐1 expression on human tumor infiltrating lymphocytes within tumors and lymphoid tissues.^82^ With regard to SPECT/CT‐based molecular imaging, several studies showed the capacity of ^111^In labeled anti‐PD‐L1 antibodies for imaging PD‐L1 expression in triple‐negative breast cancer (TNBC) and NSCLC.^73^, ^75^, ^76^ Furthermore, Pang et al demonstrated that ^131^I‐PD‐L1 monoclonal antibody (mAb) could be a potential tool for noninvasively visualizing PD‐L1 expression in primary tumors and metastases.^78^ More recently, Bensch et al conducted a first‐in‐human study to assess the potential of ^89^Zr‐atezolizumab in predicting the treatment response to PD‐L1 blockade in 22 patients across three tumors types (bladder cancer, NSCLC, and TNBC).^83^ They firstly imaged PD‐L1 status across inflammatory tissues and normal lymphoid tissues in human patients and found that the uptake of tracers before treatment seemed to be a robust biomarker to predict clinical responses and prognosis of patients.

Despite the tremendous potential of antibody‐based tracers, they have slow blood clearance that leads to long waiting time (eg, several days) between imaging scan and tracer injection, which prevents the clinical transformation of the molecular imaging. In this setting, a number of protein‐ or peptide‐based radiotracers have been fabricated to compensate the pitfalls of the antibody‐based probes.^84^, ^85^, ^86^, ^87^, ^88^, ^89^, ^90^, ^91^ For example, Trotte et al developed a ^18^F‐labeled affibody ligand that can effectively detect PD‐L1 expression in xenograft tumors by PET imaging, with preferable specificity, fast blood clearance, and low normal tissue uptake except nephridia.^85^ Larimer et al demonstrated that granzyme B PET imaging might serve as an effective biomarker for predicting responses to immunotherapy in human tumor xenograft models.^89^, ^90^

In summary, molecular imaging demonstrated enormous potential to produce a large number of biomarkers. Visualization of PD‐L1 expression in vivo is a hot spot in the latest researches on molecular imaging in immunotherapy. Although PD‐L1 status alone is insufficient to select the potential responders, it could not be ignored in some cases, such as the first‐line treatment of NSCLC. Compared with immunohistochemical technique, molecular imaging can provide a longitudinal monitoring of PD‐L1 status and reduce missed diagnosis due to small biopsy samples by assessing the level of PD‐L1 throughout the whole body.^92^ However, problems including high costs, complexity of skills, limited availability of radiotracers, lack of standardized procedures, and quality testing remain to be solved before these biomarkers can be successfully applied in clinical practice.^52^ Table 4 summarizes the emerging imaging‐based biomarkers in the latest published literatures, which are likely to provide some fresh perspectives to researchers who focus on the latest advances of medical imaging in cancer immunotherapy.

**Table 4:** Emerging imaging‐based biomarkers for immune checkpoint blockade therapy

Imaging modalities	Techniques	Biomarkers	Clinical endpoints/purposes	ICB therapy	Tumor type
Anatomic imaging	CT	≤20% increase of TB per irRECIST^22^	OS	anti‐PD‐1	Melanoma
		Tumor burden per RECIST1.1 >9 cm^55^	OS	anti‐PD‐1/‐L1	Multiple tumor types
		Tumor size per irRC and tumor density per CHOI criteria^40^	OS	anti‐PD‐1	Melanoma
Metabolic imaging	^18^F‐FDG PET/CT	Tumor metabolic response per PEPRIT^57^	BOR at ≥4 mo	anti‐CTLA‐4, anti‐PD‐1/‐L1	Melanoma
		Tumor metabolic response per PERCIST^60^	OS	anti‐CTLA‐4	Melanoma
		Tumor metabolic response per PERCIST^58^	TR at 1 mo; PFS, OS	anti‐PD‐1	NSCLC
		Whole‐body metabolic tumor volume^61^	OS	anti‐CTLA‐4	Melanoma
Functional imaging	MR‐DWI	Intermediate ADC volumes of interest after 6 mo of therapy initiation^65^	Survival time ≥5 mo	anti‐PD‐1	Glioblastoma
Radiomics	CT	Radiomics signatures with eight variables^15^	OR, SD; OS	anti‐PD‐1/‐L1	Mixed solid tumors
		Clinical‐radiomic models^68^	TTP <2 mo; HPD	anti‐CTLA‐4, anti‐PD‐1/‐L1	NSCLC
		Tumor skewness at coarse texture scale^67^	OS, PFS	anti‐PD‐1	Melanoma
Molecular imaging	PET	^89^Zr‐desferrioxamine‐labeled anti‐CD8 cys‐diabody^71^	TR	anti‐PD‐L1	HTXM‐MST
		^89^Zr‐labeled PEGylated single‐domain antibody fragments^72^	TR	anti‐CTLA‐4	HTXM‐MST
		^64^Cu‐, ^89^Zr‐labeled antibodies^74^, ^77^, ^79^	PD‐L1 expression	—	HTXM‐MST
		^89^Zr‐, ^64^Cu‐labeled antibodies^81^, ^82^	PD‐1 expression	—	HTXM‐MST
		^89^Zr‐labeled antibody^83^	TR; PFS, OS	anti‐PD‐L1	Human patients‐MST
		^64^Cu‐, ^18^F‐ labeled peptide 84, 91	PD‐L1 expression	—	HTXM‐MST
		^18^F‐, ^64^Cu‐, ^68^Ga‐labeled protein^85^, ^87^, ^88^	PD‐L1 expression	—	HTXM‐MST
		^68^Ga‐NOTA‐GZP^89^, ^90^	TR; granzyme B expression	anti‐PD‐1, anti‐CTLA‐4	HTXM‐MST
	NIRF/PET	Liposome‐doxorubicin‐^64^Cu/IRDye800CW‐labeled antibody^80^	PD‐1 expression	—	HTXM‐breast cancer
	NIRF and MRI	Nanohybrid liposomal cerasome nanoparticles^86^	PD‐L1 expression	—	HTXM‐MST
	SPECT/CT	^111^In‐, ^131^I‐labeled antibodies^73^, ^75^, ^76^, ^78^	PD‐L1 expression	—	HTXM‐MST

Abbreviations: ADC, apparent diffusion coefficient; BOR, best overall response; CT, computed tomography; DWI, diffusion‐weighted imaging; ^18^F‐FDG PET, 18F‐fluorodeoxyglucose positron emission tomography; GZP, granzyme B specific PET imaging agent; HTXM, human tumor xenograft models; ICB, immune checkpoint blockade; MR, magnetic resonance; MST, mixed solid tumors; NIRF, near‐infrared fluorescence; NSCLC, non‐small cell lung cancer; OR, objective response; OS, overall survival; PEPRIT, PET/CT criteria for early prediction of response to ICI therapy; PERCIST, PET response criteria in solid tumors; PFS, progression‐free survival; SD, stable disease; SPECT, single‐photon emission computed tomography; TR, treatment response; TTP, time to progression.

## 5 CONCLUSIONS

The introduction of ICB has dramatically improved clinical outcomes of advanced cancer patients spanning various tumor types. Three unconventional response patterns in immunotherapy including pseudoprogression, dissociated response, and hyperprogression are accompanied with distinguished prognosis and pose significant challenges to response assessment in immunotherapy. Medical imaging is the cornerstone for longitudinal monitoring of the changes in tumor burden. Noninvasive imaging studies play crucial roles in detection and surveillance of irAEs. A wide range of imaging biomarkers including anatomic, metabolic, and functional parameters, radiomic features, and molecular probes have demonstrated enormous potential for predicting responses and prognosis in patients receiving ICB therapy. Further efforts are warranted to achieve the clinical transformation of these predictive imaging biomarkers for optimizing therapeutic strategies and improving patient outcomes in clinical practice.

## CONFLICT OF INTEREST

The authors declare no conflict of interest.
